# Marked and reversible circulating insulin-like growth factor-1 elevation during teprotumumab N01 treatment for thyroid eye disease with limited correspondence to glycemic changes

**DOI:** 10.3389/fendo.2026.1870933

**Published:** 2026-07-15

**Authors:** Wei Zhao, Lingli Zhou, Ying Gao, Xianghai Zhou, Xueyao Han, Xiuying Zhang, Linong Ji

**Affiliations:** 1 Department of Endocrinology and Metabolism, Peking University People’s Hospital, Beijing, China; 2 Peking University Diabetes Center, Beijing, China

**Keywords:** glycemic changes, hyperglycemia, insulin-like growth factor-1, insulin-like growth factor-1 receptor inhibitor, teprotumumab N01, thyroid eye disease

## Abstract

**Background:**

Insulin-like growth factor-1 (IGF-1) receptor (IGF-1R) inhibitors have changed the treatment landscape for moderate-to-severe thyroid eye disease (TED), but the longitudinal behavior of circulating IGF-1 during and after therapy remains insufficiently characterized. This study aimed to describe serum IGF-1 dynamics in patients with TED treated with IGF-1R inhibitor teprotumumab N01 and to explore their relationship with glycemic changes.

**Methods:**

In this retrospective cohort study, 92 patients with moderate-to-severe TED treated with teprotumumab N01 who had longitudinal IGF-1 measurements were included. IGF-1 dynamics were characterized at infusion-based visits and during post-treatment follow-up. Glycemic changes were assessed using fasting blood glucose (FBG), hemoglobin A1c (HbA1c), and glycated albumin (GA). Patients were classified by baseline glycemic status. Associations between IGF-1 metrics and glycemic changes were evaluated using multivariable linear regression for continuous glycemic outcomes and logistic regression for threshold-defined glycemic events, with sequential adjustment for age, sex, baseline glycemic markers, and baseline IGF-1 when applicable. Additional analyses were performed according to baseline glycemic status and baseline HbA1c quartiles.

**Results:**

Baseline serum IGF-1 was 151.0 ng/mL (IQR 116.8–187.3). IGF-1 increased markedly after treatment initiation, with a median early-treatment peak fold change of 3.5 (IQR 3.0–4.3) and a median on-treatment peak concentration of 649.5 ng/mL (IQR 520.8–753.8), corresponding to a 4.2-fold increase from baseline (IQR 3.5–5.0). IGF-1 remained elevated during the first 3 months after the last infusion and then declined progressively, generally approaching baseline by 6–9 months or later. Glycemic markers showed modest increases, with median peak increases from baseline of 0.40% (IQR 0.20–0.77) for HbA1c, 1.63% (IQR 0.95–2.39) for GA, and 0.65 mmol/L (IQR 0.30–1.14) for FBG. Glycemic deterioration was most pronounced in patients with baseline dysglycemia. In contrast, neither baseline IGF-1 nor IGF-1 dynamic metrics were consistently identified as independent correlates of glycemic changes after adjustment for age, sex, and baseline glycemic markers and IGF-1. Similar findings were observed in subgroup and HbA1c quartile analyses.

**Conclusion:**

Teprotumumab N01 treatment was associated with a substantial, early, and broadly reversible increase in circulating IGF-1 in patients with moderate-to-severe TED, but IGF-1 dynamics did not serve as an independent indicator of glycemic deterioration.

## Introduction

Thyroid eye disease (TED) is an autoimmune orbital disorder that can cause proptosis, eyelid retraction, diplopia, and impaired quality of life (1). The clinical use of insulin-like growth factor-1 (IGF-1) receptor (IGF-1R) inhibitors has substantially changed the treatment landscape for moderate-to-severe TED (2). Teprotumumab has shown clinically meaningful efficacy in randomized trials (3, 4), and subsequent studies have extended this evidence to patients with longer disease duration, lower disease activity, and Asian populations, including Chinese patients treated with teprotumumab N01, formerly known as IBI311 (5–7).

The therapeutic rationale for IGF-1R blockade is supported by experimental and translational evidence showing that IGF-1R signaling contributes to orbital fibroblast activation and interacts with thyrotropin receptor (TSHR)-related pathways in TED (8, 9). Pharmacokinetic modeling of teprotumumab has suggested sustained target engagement across the recommended dosing interval (10). In addition, studies of IGF-1R inhibitors have reported treatment-related increases in circulating IGF-1, suggesting that serum IGF-1 may change substantially during receptor inhibition (11, 12). However, the longitudinal pattern of serum IGF-1 during and after IGF-1R inhibitor therapy for TED remains insufficiently characterized.

This question is clinically relevant because the IGF-1 axis is closely connected to systemic endocrine and metabolic regulation. IGF-1 signaling overlaps with insulin receptor pathways and may influence glucose metabolism (13, 14). Hyperglycemia and worsening glycemic markers have been reported during IGF-1R inhibitor therapy, particularly in patients with baseline metabolic vulnerability (15, 16). It remains unclear whether circulating IGF-1 dynamics provide additional information about glycemic changes during IGF-1R inhibition or represent a distinct treatment-related laboratory pattern.

Therefore, we conducted a retrospective cohort study of patients with moderate-to-severe TED treated with teprotumumab N01. We aimed to characterize the timing, magnitude, and reversibility of circulating IGF-1 changes during and after treatment, and to explore their relationship with glycemic changes, including fasting blood glucose (FBG), hemoglobin A1c (HbA1c), and glycated albumin (GA).

## Materials and methods

### Study design and participants

**Figure 1:**
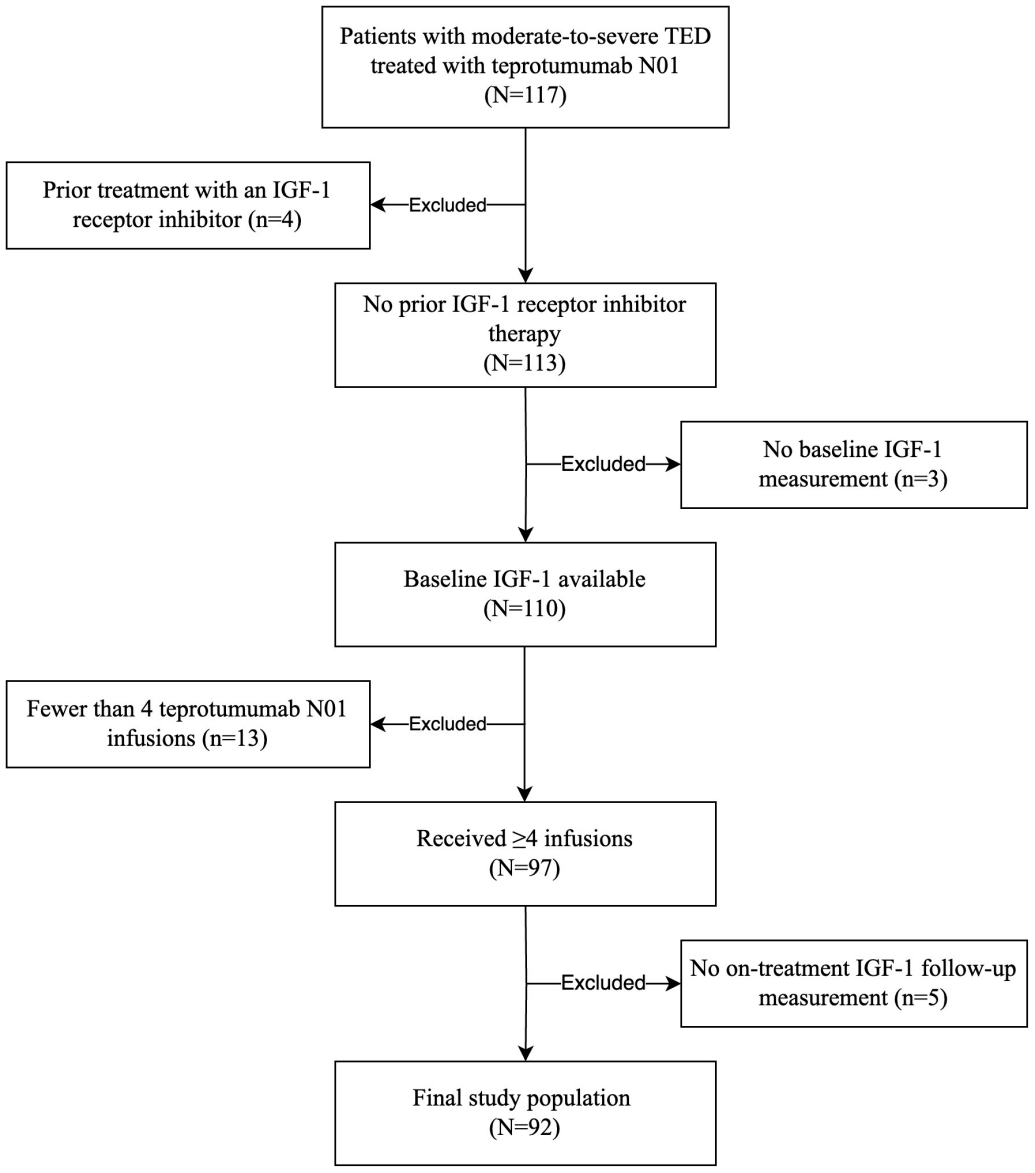
Flow diagram of patient inclusion. IGF-1, insulin-like growth factor-1; TED, thyroid eye disease.

This retrospective cohort study included patients with moderate-to-severe TED who were treated with teprotumumab N01 at Peking University People’s Hospital between June 2023 and March 2026. Clinical and laboratory data were extracted from medical records. Patients were excluded if they had received prior treatment with an IGF-1 receptor inhibitor before the current treatment course, received fewer than four teprotumumab N01 infusions, lacked a baseline IGF-1 measurement, or had insufficient IGF-1 follow-up measurement. The final analytic cohort therefore included patients with both baseline and follow-up IGF-1 measurements, as shown in **Figure 1**.

The study was approved by the Ethics Committee of Peking University People’s Hospital. The requirement for informed consent was waived according to the retrospective nature of the study.

### Treatment and laboratory assessments

Teprotumumab N01 was administered as an initial dose of 10 mg/kg followed by 20 mg/kg for subsequent infusions, administered at intervals of approximately 3–4 weeks for up to eight infusions. Laboratory assessments, including serum IGF-1 and glycemic markers, were generally performed before each scheduled infusion.

Baseline was defined as the pre-infusion assessment before the first infusion. The early-treatment period was defined as assessments before the second or third infusion. The on-treatment period was defined as pre-infusion assessments during treatment; measurements obtained within 6 weeks after the last infusion were considered peri-treatment and included in the on-treatment analysis. Post-treatment follow-up measurements were categorized according to months after the last infusion: 0–1, 1–3, 3–6, 6–9, and ≥9 months.

Serum IGF-1 was measured using the IMMULITE 2000 IGF-I assay (Siemens Healthcare Diagnostics Products Limited, United Kingdom), a solid-phase, enzyme-labeled chemiluminescent immunometric assay. Results were reported in ng/mL. The analytical measuring range of the assay was 15–1000 ng/mL. Specimens exceeding the upper detection limit were manually diluted and reanalyzed, with final IGF-1 concentrations corrected for the dilution factor.

IGF-1 fold change was calculated as the ratio of the IGF-1 value to baseline IGF-1. The infusion number at peak IGF-1 was defined as the infusion-based assessment number at which the individual on-treatment peak IGF-1 was observed. The first infusion-based assessment at which IGF-1 reached at least 80% of the individual on treatment peak was also recorded.

### Baseline glycemic status and glycemic outcomes

Baseline glycemic status was classified using documented diabetes history and available baseline HbA1c and FBG values according to standard glycemic thresholds (17). Patients with documented diabetes history, baseline HbA1c ≥6.5%, or baseline FBG ≥7.0 mmol/L were classified as diabetes. Patients without diabetes history but with baseline HbA1c 5.7–6.4% or FBG 5.6–6.9 mmol/L were classified as prediabetes. Patients with available baseline HbA1c or FBG values below these thresholds were classified as having normoglycemia.

Early glycemic changes were defined as changes from baseline to the early-treatment peak measured at the pre-infusion assessments before the second or third infusion. On-treatment peak glycemic changes were defined as changes from baseline to the highest value observed during treatment or within 6 weeks after the last infusion. Continuous glycemic outcomes included early and on-treatment peak changes in HbA1c, GA, and FBG. Threshold-defined glycemic events included peak HbA1c ≥6.5%, peak HbA1c increase ≥0.5%, peak GA ≥16%, and peak GA increase ≥2%.

### Statistical analysis

Continuous variables were summarized as mean ± standard deviation or median (interquartile range, IQR), as appropriate, and categorical variables as number (percentage). Comparisons across baseline glycemic status categories were performed using the Kruskal–Wallis test for continuous variables and the chi-square test or Fisher’s exact test for categorical variables.

Associations between IGF-1 metrics and continuous glycemic changes were evaluated using multivariable linear regression. IGF-1 exposure variables included baseline IGF-1, early-treatment peak IGF-1 fold change, and on-treatment peak IGF-1 fold change. Early glycemic outcomes were analyzed in relation to baseline IGF-1 and early-treatment IGF-1 fold change, whereas on-treatment peak glycemic outcomes were analyzed in relation to baseline IGF-1, early-treatment IGF-1 fold change, and on-treatment peak IGF-1 fold change. Continuous exposure and outcome variables were standardized before modeling, and results are reported as standardized β coefficients with 95% confidence intervals.

Three sequential models were used: Model 1 adjusted for age and sex; Model 2 additionally adjusted for the corresponding baseline glycemic marker; and Model 3 additionally adjusted for baseline IGF-1 when the IGF-1 exposure variable was not baseline IGF-1 itself. The corresponding baseline glycemic marker was baseline HbA1c for HbA1c outcomes, baseline GA for GA outcomes, and baseline FBG for FBG outcomes. Logistic regression using the same adjustment strategy was performed as a supplementary analysis for threshold-defined glycemic events.

To examine whether associations differed by baseline glycemic vulnerability, analyses were repeated in baseline normoglycemia and dysglycemia subgroups, with dysglycemia defined as prediabetes or diabetes categories at baseline. In exploratory HbA1c quartile analyses, patients without documented diabetes history were stratified by baseline HbA1c quartiles, and descriptive and Model 3 regression analyses were performed. Regression analyses used complete cases for variables included in each model, and missing data were not imputed. All analyses were exploratory, and *P* values were nominal without adjustment for multiple comparisons. Statistical analyses and visualization were performed using Python 3.11.8.

## Results

### Study population and baseline characteristics

A total of 117 moderate-to-severe TED patients were screened, and 92 patients were included in the final analytic cohort (**Figure 1**). The cohort included 55 females and 37 males, with a mean age of 44.1 years. The median duration of TED was 12.5 months. At baseline, the median serum IGF-1 was 151.0 ng/mL (IQR 116.8–187.3). Baseline FBG, HbA1c, and GA had median values of 4.87 mmol/L (IQR 4.61–5.17), 5.60% (IQR 5.40–5.90), and 12.69% (IQR 12.13–13.33), respectively (**Table 1**).

**Table 1:** Baseline characteristics.

Characteristic	Overall (N = 92)	Female (N = 55)	Male (N = 37)
Age (years)	44.1 ± 12.1	42.6 ± 12.2	46.4 ± 11.7
Smoking history [n (%)]	33 (35.9)	7 (12.7)	26 (70.3)
TED duration (months)	12.5 (7.7, 22.7)	11.7 (7.3, 21.3)	14.8 (9.3, 23.2)
Proptosis (mm)			
Right eye	22.0 (21.0, 25.0)	22.0 (20.1, 23.8)	24.0 (22.0, 25.0)
Left eye	23.0 (21.0, 25.1)	22.0 (20.6, 24.0)	24.0 (23.0, 25.9)
CAS			
Right eye	3.0 (2.0, 4.0)	3.0 (1.5, 4.0)	3.5 (2.0, 4.0)
Left eye	3.0 (1.0, 4.0)	3.0 (1.0, 3.0)	4.0 (2.0, 4.0)
Treatment exposure [n (%)]			
4 infusions	33 (35.9)	24 (43.6)	9 (24.3)
5–7 infusions	8 (8.7)	3 (5.5)	5 (13.5)
8 infusions	51 (55.4)	28 (50.9)	23 (62.2)
IGF-1 (ng/mL)	151.0 (116.8, 187.3)	145.0 (120.0, 183.0)	155.0 (116.0, 205.0)
History of diabetes [n (%)]	5 (5.4)	0 (0)	5 (13.5)
FBG (mmol/L)	4.87 (4.61, 5.17)	4.83 (4.63, 5.15)	4.88 (4.58, 5.54)
HbA1c (%)	5.60 (5.40, 5.90)	5.60 (5.40, 5.83)	5.80 (5.60, 6.08)
GA (%)	12.69 (12.13, 13.33)	12.70 (12.13, 13.33)	12.68 (12.10, 13.40)
FT3 (pmol/L)	5.22 (4.80, 5.67)	4.93 (4.56, 5.31)	5.52 (5.26, 6.17)
FT4 (pmol/L)	16.58 (14.32, 19.17)	15.42 (13.73, 18.66)	17.60 (16.47, 19.67)
TSH (µIU/mL)	1.46 (0.37, 2.86)	1.54 (0.36, 3.56)	1.15 (0.41, 2.59)
TRAb (IU/L)	4.05 (1.62, 10.42)	4.75 (1.79, 10.43)	3.77 (1.56, 10.29)
TSI (IU/L)	2.84 (1.04, 6.28)	3.09 (1.38, 6.26)	1.93 (0.95, 7.08)

Data are presented as mean ± SD, median (IQR), or number (%), as appropriate. CAS, clinical activity score; FBG, fasting blood glucose; FT3, free triiodothyronine; FT4, free thyroxine; GA, glycated albumin; HbA1c, glycated hemoglobin; IGF-1, insulin-like growth factor-1; TED, thyroid eye disease; TRAb, thyrotropin receptor antibody; TSH, thyroid-stimulating hormone; TSI, thyroid-stimulating immunoglobulin.

### IGF-1 dynamics on- and post-treatment

**Figure 2:**
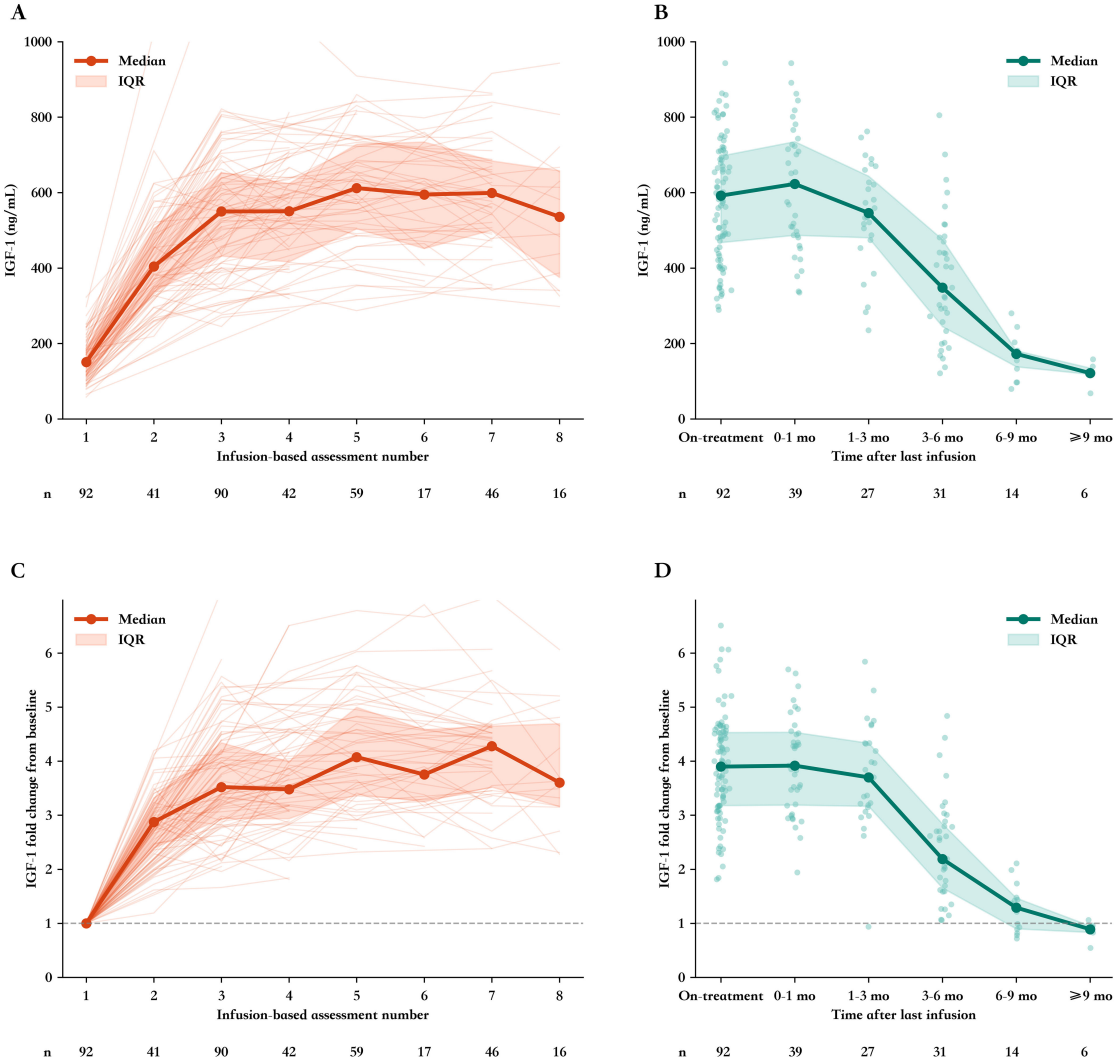
Longitudinal serum IGF-1 dynamics during and after teprotumumab N01 treatment. **(A)** Serum IGF-1 concentrations during treatment by infusion-based assessment number. **(B)** Serum IGF-1 concentrations at the last on-treatment assessment and during post-treatment follow-up. **(C)** IGF-1 fold change from baseline during treatment. **(D)** IGF-1 fold change from baseline at the last on-treatment assessment and during post-treatment follow-up. Lines and points indicate medians, shaded areas indicate interquartile ranges, and faint individual lines or jittered points show patient-level observations. The dashed horizontal line in **(C, D)** indicates a fold change of 1.0 from baseline. The numbers below the x-axis indicate the number of available data at each timepoint. IGF-1, insulin-like growth factor-1; IQR, interquartile range.

IGF-1 increased markedly after treatment initiation (**Figure 2**). The median on-treatment peak IGF-1 concentration was 649.5 ng/mL (IQR 520.8–753.8), corresponding to a median fold change of 4.2 (IQR 3.5–5.0). Nearly all patients had a peak IGF-1 level at least 2 times the baseline level, nearly 90% reached at least 3 times baseline, and 25% reached at least 5 times baseline. The median infusion-based assessment number at peak IGF-1 was 5 (IQR 4–8). However, IGF-1 increased rapidly early during treatment, with the first assessment reaching at least 80% of the individual peak occurring at a median of the third infusion. During the early-treatment period, the median peak IGF-1 concentration was 557.0 ng/mL (IQR 436.0–660.8), corresponding to a median fold change of 3.5 (IQR 3.0–4.3; **Table 2**).

**Table 2:** IGF-1 dynamics and glycemic outcomes during IGF-1R inhibitor therapy.

Indicator	Time/window	Metric	No.	Value
IGF-1	On treatment	Peak value (ng/mL)	92	649.5 (520.8, 753.8)
		Peak fold change	92	4.2 (3.5, 5.0)
		≥2-fold [n (%)]	92	91 (98.9)
		≥3-fold [n (%)]	92	82 (89.1)
		≥4-fold [n (%)]	92	51 (55.4)
		≥5-fold [n (%)]	92	23 (25.0)
		Infusion No. at peak	92	5 (4, 8)
		First infusion No. reaching ≥80% peak	92	3 (3, 4)
	Early treatment	Peak value (ng/mL)	90	557.0 (436.0, 660.8)
		Peak fold change	90	3.5 (3.0, 4.3)
HbA1c	On treatment	Peak value (%)	77	6.00 (5.70, 6.60)
		Peak value ≥6.5% [n (%)]	77	22 (28.6)
		Peak change from baseline (%)	74	0.40 (0.20, 0.77)
		Peak change ≥0.5% [n (%)]	74	32 (43.2)
GA	On treatment	Peak value (%)	80	14.26 (13.44, 15.50)
		Peak value ≥16% [n (%)]	80	17 (21.2)
		Peak change from baseline (%)	78	1.63 (0.95, 2.39)
		Peak change ≥2% [n (%)]	78	29 (37.2)
	Early treatment	Peak value (%)	77	13.69 (13.12, 14.70)
		Peak change from baseline (%)	76	1.04 (0.59, 1.41)
FBG	On treatment	Peak value (mmol/L)	91	5.50 (5.08, 6.29)
		Peak change from baseline (mmol/L)	90	0.65 (0.30, 1.14)
	Early treatment	Peak value (mmol/L)	91	5.14 (4.79, 5.79)
		Peak change from baseline (mmol/L)	90	0.35 (-0.07, 0.77)

Data are presented as median (IQR) or number (%), as appropriate. Early treatment was defined as pre-infusion assessments at the second or third infusion. On-treatment peak was defined as the highest value during pre-infusion assessments and peri-treatment measurements obtained within 6 weeks after the last infusion. Infusion number refers to the pre-infusion assessment number. The first infusion number reaching ≥80% peak was the earliest assessment at which IGF-1 reached at least 80% of the individual on-treatment peak. FBG, fasting blood glucose; GA, glycated albumin; HbA1c, glycated hemoglobin; IGF-1, insulin-like growth factor-1.

Post-treatment IGF-1 measurements showed a gradual decline (**Figure 2**). IGF-1 remained elevated during the first 3 months after the last infusions but decreased progressively thereafter. By 6–9 months and later, IGF-1 levels approached baseline levels, with a similar pattern observed for IGF-1 fold change from baseline.

**Figure 3:**
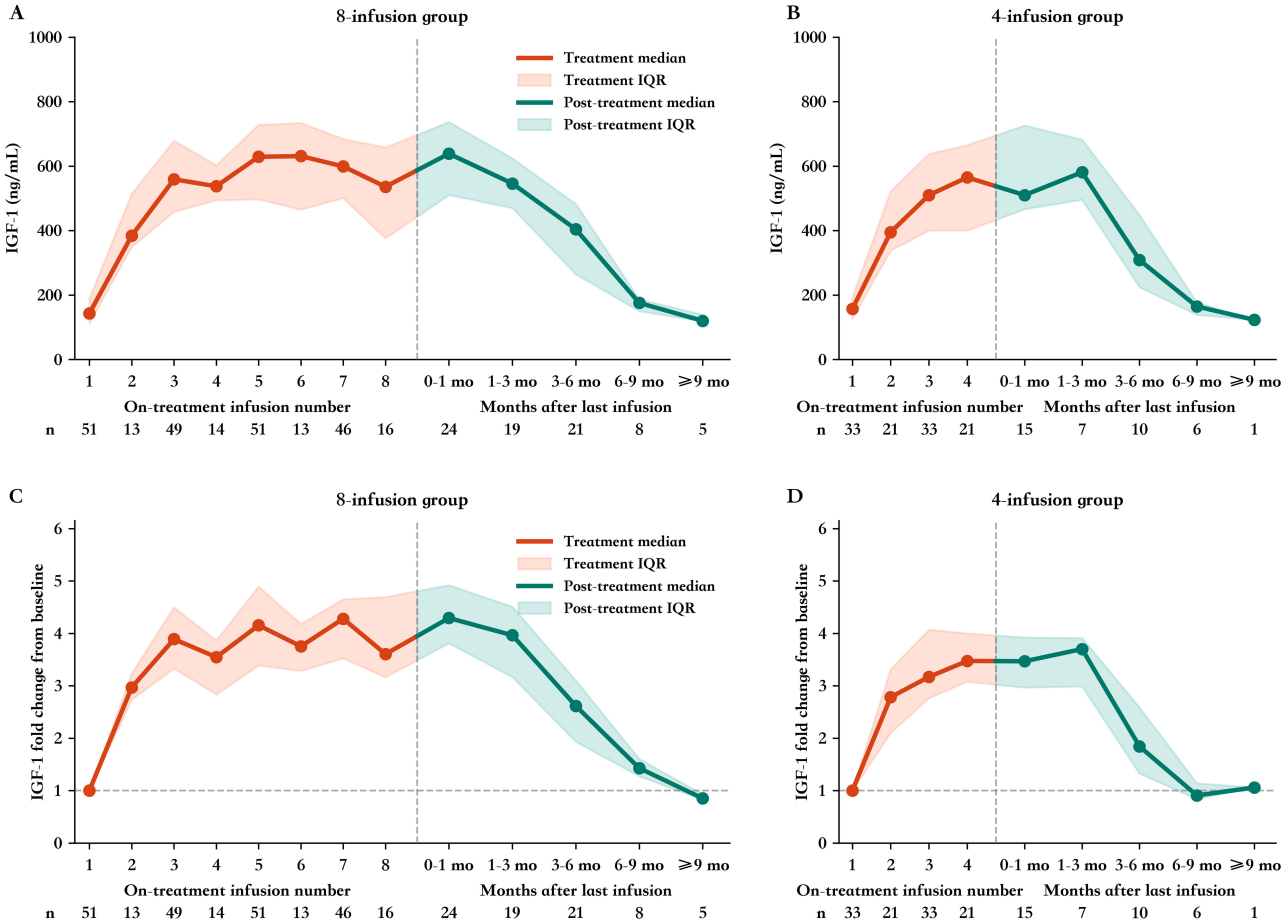
Serum IGF-1 dynamics stratified by treatment exposure. **(A, B)** Serum IGF-1 concentrations in patients receiving **(A)** 8 infusions and **(B)** 4 infusions of teprotumumab N01. **(C, D)** IGF-1 fold change from baseline in patients receiving **(C)** 8 infusions and **(D)** 4 infusions. Lines and shaded areas indicate the median and interquartile range. The horizontal dashed line in panels C and D indicates a fold change of 1.0. The numbers below the x-axis indicate the number of patients with available data at each timepoint. IGF-1, insulin-like growth factor-1; IQR, interquartile range.

When stratified by treatment exposure, IGF-1 increased rapidly in both the 4-infusion and 8-infusion groups, with marked elevation observed before the third infusion (**Figure 3**). In both groups, IGF-1 remained elevated during treatment and persisted during the first 3 months after the last infusion, followed by a progressive decline thereafter. Similar patterns were observed for IGF-1 fold change from baseline.

Sex- and age-stratified analyses showed similar trajectories. Though differences occurred in absolute IGF-1 concentrations, particularly across age groups, the fold-change trajectories from baseline were broadly similar across strata (**Supplementary Figures 1**–**S3**). Post-treatment decline in IGF-1 fold change was observed consistently across sex and age subgroups.

### Glycemic outcomes and baseline glycemic vulnerability

Overall, glycemic markers showed modest increases during treatment (**Table 2**). The median on-treatment peak HbA1c was 6.00% (IQR 5.70–6.60), with 28.6% of patients reaching a peak HbA1c of ≥6.5%. The median peak increase from baseline in HbA1c was 0.40% (IQR 0.20–0.77), corresponding to 43.2% of patients reaching an increase of ≥0.5%. Similar increases were observed in GA, with a median peak increase of 1.63% (IQR 0.95–2.39), and 37.2% of patients reached an increase of ≥2%. The median on-treatment peak FBG was 5.50 mmol/L (IQR 5.08–6.29), with a median increase of 0.65 mmol/L (IQR 0.30–1.14). Early-treatment increases were already observed for both GA and FBG, with median increases from baseline of 1.04% and 0.35 mmol/L, respectively, although the absolute values generally remained below abnormal thresholds (**Supplementary Figure 4**).

**Figure 4:**
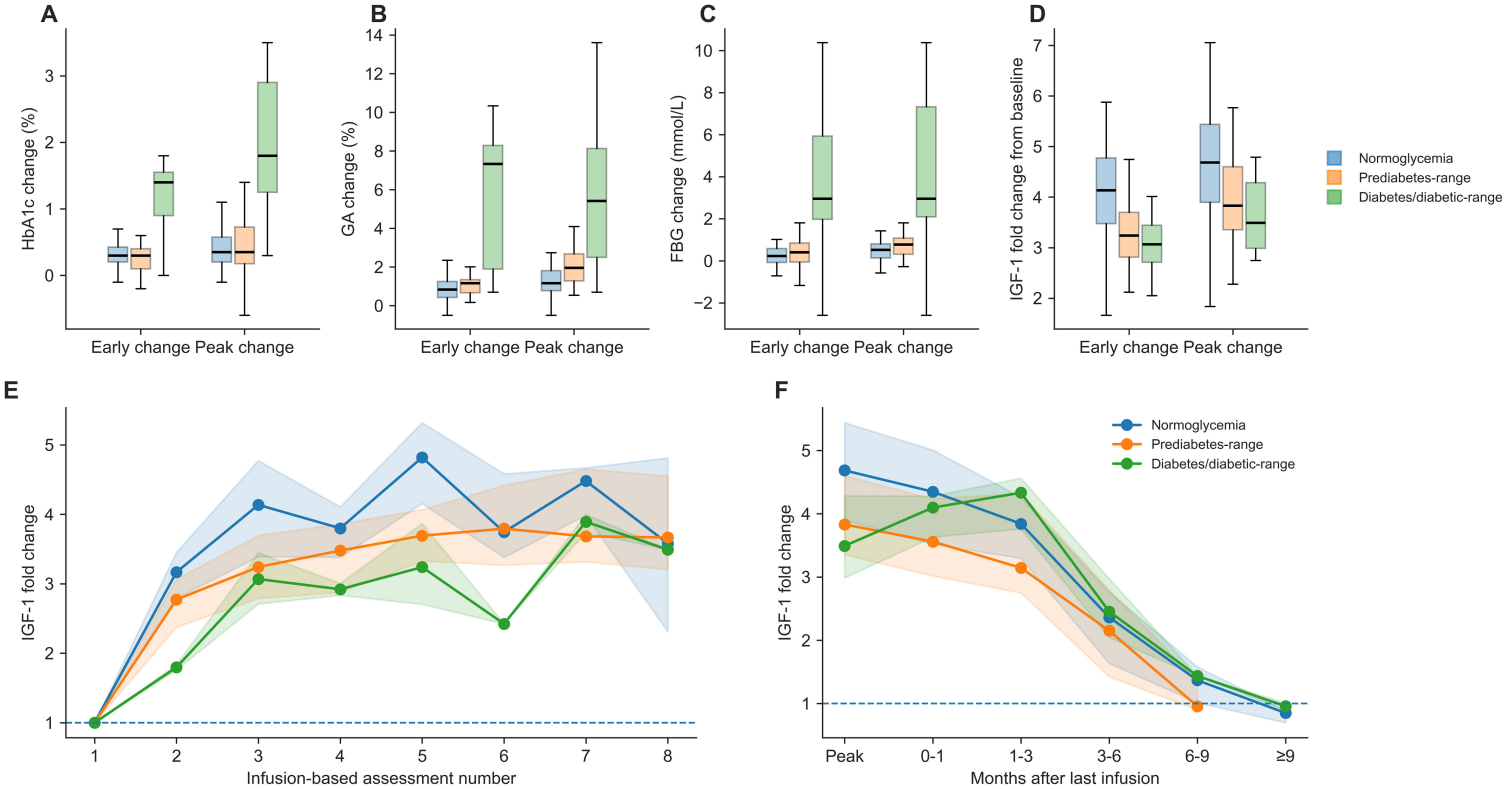
Glycemic changes and IGF-1 dynamics according to baseline glycemic status. **(A–C)** Early-treatment and on-treatment peak changes in HbA1c, GA, and FBG according to baseline glycemic status. **(D)** Early-treatment and on-treatment peak IGF-1 fold changes according to baseline glycemic status. **(E)** Treatment-period IGF-1 fold-change trajectories stratified by baseline glycemic status. **(F)** Post-treatment IGF-1 fold-change trajectories stratified by baseline glycemic status, with the first time point indicating the individual on-treatment peak. Lines indicate medians, shaded areas indicate interquartile ranges, and boxplots show medians and interquartile ranges. FBG, fasting blood glucose; GA, glycated albumin; HbA1c, hemoglobin A1c; IGF-1, insulin-like growth factor-1.

We next examined glycemic outcomes according to baseline glycemic status. Among the 92 included patients, 44 were classified as having baseline normoglycemia, 37 as having prediabetes, and 9 as categorized as diabetes; 2 patients were not classified because of missing baseline HbA1c and FBG values. Glycemic worsening was most pronounced in patients with baseline diabetes category and was already evident during the early-treatment period. In this group, early changes in FBG, HbA1c, and GA reached 2.96 mmol/L (IQR 1.98–5.93), 1.40% (IQR 0.90–1.55), and 7.33% (IQR 1.89–8.28), respectively. This pattern persisted for on-treatment peak changes, with peak changes in FBG, HbA1c, and GA of 2.96 mmol/L (IQR 2.09–7.33), 1.80% (IQR 1.25–2.90), and 5.42% (IQR 2.50–8.13), respectively. In contrast, changes were relatively modest in the normoglycemia and prediabetes groups (**Table 3**; **Figures 4A–C**).

**Table 3:** Baseline glycemic status-stratified IGF-1 dynamics and glycemic changes during IGF-1R inhibitor therapy.

Characteristic	Normoglycemia (N = 44)	Prediabetes (N = 37)	Diabetes (N = 9)	P value
Age (years)	40.0 (32.0, 45.0)	45.0 (36.0, 55.0)	56.0 (48.0, 63.0)	0.005
Male sex [n (%)]	12 (27.3)	18 (48.6)	7 (77.8)	0.009
Baseline FBG (mmol/L)	4.72 (4.59, 4.98)	4.94 (4.66, 5.54)	6.65 (5.16, 7.07)	0.003
Baseline HbA1c (%)	5.45 (5.30, 5.60)	5.90 (5.70, 6.00)	6.50 (6.50, 6.80)	<0.001
Baseline GA (%)	12.64 (12.00, 13.30)	12.73 (12.22, 13.43)	13.49 (12.26, 15.42)	0.463
Baseline IGF-1 (ng/mL)	145.0 (114.8, 184.8)	152.0 (127.0, 187.0)	151.0 (126.0, 191.0)	0.942
Early IGF-1 fold change	4.1 (3.5, 4.8)	3.2 (2.8, 3.7)	3.1 (2.7, 3.4)	<0.001
Peak IGF-1 fold change	4.7 (3.9, 5.4)	3.8 (3.4, 4.6)	3.5 (3.0, 4.3)	0.001
Early FBG change (mmol/L)	0.23 (-0.08, 0.58)	0.41 (-0.06, 0.85)	2.96 (1.98, 5.93)	0.009
Early HbA1c change (%)	0.30 (0.20, 0.43)	0.30 (0.10, 0.40)	1.40 (0.90, 1.55)	0.009
Early GA change (%)	0.83 (0.43, 1.25)	1.16 (0.67, 1.34)	7.33 (1.89, 8.28)	0.002
Peak FBG change (mmol/L)	0.53 (0.14, 0.81)	0.78 (0.31, 1.08)	2.96 (2.09, 7.33)	<0.001
Peak HbA1c change (%)	0.35 (0.20, 0.58)	0.35 (0.17, 0.73)	1.80 (1.25, 2.90)	0.002
Peak change ≥0.5% [n/N (%)]	13/38 (34.2)	12/28 (42.9)	7/8 (87.5)	0.022
Peak GA change (%)	1.17 (0.77, 1.81)	1.96 (1.28, 2.67)	5.42 (2.50, 8.13)	<0.001
Peak change ≥2% [n/N (%)]	7/39 (17.9%)	15/30 (50.0%)	7/8 (87.5%)	<0.001

Data are shown according to baseline glycemic status, classified using documented diabetes history and available baseline HbA1c and FBG values. Continuous variables are presented as median (interquartile range), and categorical variables as n (%). P values were calculated using the Kruskal–Wallis test for continuous variables and the chi-square or Fisher’s exact test for categorical variables, as appropriate. Early changes were defined as changes from baseline to the early-treatment peak before the second or third infusion, whereas peak changes were defined as changes from baseline to the on-treatment peak during treatment or within 6 weeks after the last infusion. FBG, fasting blood glucose; GA, glycated albumin; HbA1c, hemoglobin A1c; IGF-1, insulin-like growth factor-1.

### Association between IGF-1 dynamics and glycemic outcomes

Baseline IGF-1 concentrations were similar across baseline glycemic status categories. In contrast, treatment-related IGF-1 fold changes differed significantly and were lower in patients with greater baseline glycemic vulnerability (**Table 3**). Early-treatment peak IGF-1 fold change was 4.1 (IQR 3.5–4.8) in the normoglycemia group, 3.2 (IQR 2.8–3.7) in the prediabetes group, and 3.1 (IQR 2.7–3.4) in the diabetes group (*P* < 0.001). A similar pattern was observed for on-treatment peak IGF-1 fold change: 4.7 (IQR 3.9–5.4), 3.8 (IQR 3.4–4.6), and 3.5 (IQR 3.0–4.3), respectively (*P* = 0.001). Longitudinal IGF-1 trajectories showed similar patterns across groups, although absolute fold changes were consistently lower in metabolically vulnerable patients (**Figures 4D–F**).

**Figure 5:**
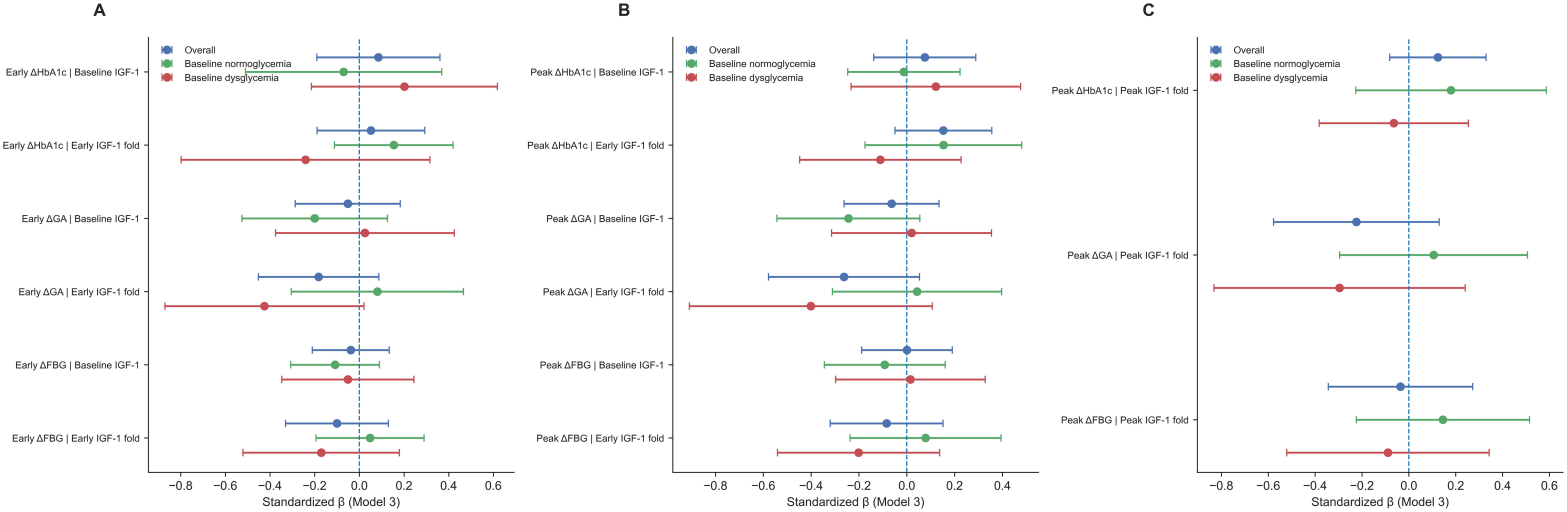
Multivariable associations of IGF-1 metrics with glycemic changes in the full cohort and baseline glycemic status subgroups. **(A)** Associations of baseline IGF-1 and early-treatment IGF-1 fold change with early glycemic changes. **(B)** Associations of baseline IGF-1 and early-treatment IGF-1 fold change with on-treatment peak glycemic changes. **(C)** Associations of on-treatment peak IGF-1 fold change with on-treatment peak glycemic changes. Forest plots show standardized β coefficients with 95% confidence intervals from multivariable linear regression models in the overall cohort and in baseline normoglycemia and dysglycemia subgroups. Models were adjusted for age, sex, and the corresponding baseline glycemic marker; baseline IGF-1 was additionally included when the IGF-1 exposure variable was not baseline IGF-1 itself. FBG, fasting blood glucose; GA, glycated albumin; HbA1c, hemoglobin A1c; IGF-1, insulin-like growth factor-1.

However, in multivariable linear regression models, these IGF-1 metrics were not consistently associated with glycemic changes after adjustment for age, sex, the corresponding baseline glycemic marker, and baseline IGF-1 when applicable (**Supplementary Table 1**). A similar pattern was observed in analyses stratified by baseline normoglycemia and dysglycemia (**Figure 5**). Logistic regression analyses for threshold-defined glycemic events also did not support a consistent independent association between IGF-1 metrics and glycemic worsening after full adjustment (**Supplementary Table 2**). In exploratory analyses among patients without diabetes history, HbA1c quartile stratification yielded similar findings. Across HbA1c quartiles, no consistent monotonic association was observed between IGF-1 fold changes and peak glycemic changes across quartiles in Model 3 analyses (**Supplementary Tables 3**, **S4**). Overall, these analyses did not support a consistent association between the magnitude or timing of IGF-1 elevation and glycemic deterioration.

## Discussion

In this retrospective cohort of patients with moderate-to-severe TED treated with teprotumumab N01, we observed a marked, early, and reversible increase in serum IGF-1 during treatment. IGF-1 rose rapidly after treatment initiation, with most patients approaching their individual on-treatment peak by the early infusion-based assessments, remained elevated during treatment and early follow-up, and then declined progressively after treatment cessation. These longitudinal data add to the limited clinical evidence describing serum IGF-1 behavior during IGF-1R inhibitor therapy for TED. Importantly, despite this substantial IGF-1 elevation, IGF-1 dynamic metrics did not show consistent explanatory value for glycemic changes after adjustment for baseline glycemic markers and other clinical covariates. Together, these findings suggest that IGF-1 elevation during IGF-1R inhibition is a prominent treatment-related laboratory pattern, but not a reliable standalone marker of glycemic risk.

The marked increase in circulating IGF-1 observed in our cohort is consistent with prior pharmacokinetic and pharmacodynamic studies of IGF-1R inhibitors. In our cohort, IGF-1 increased rapidly, with a median early-treatment peak fold change of approximately 3.5 and an on-treatment peak fold change of approximately 4.2. This magnitude is broadly comparable with prior IGF-1R inhibitor studies: early oncology data in the teprotumumab pharmacokinetic analysis showed serum IGF-I increase of approximately 100–350% after drug exposure (10, 11), figitumumab increased total IGF-1 by approximately 4- to 5-fold in healthy participants (12), and ganitumab was associated with a more than 2-fold increase in mean IGF-1 in a phase I study (18). A recent small report in TED patients has also suggested that IGF-1 may remain elevated for several months after teprotumumab treatment, but systematic longitudinal evidence remains limited (19). Together, these findings support circulating IGF-1 elevation as a recurring treatment-related pattern during IGF-1R blockade, although direct numerical comparisons across studies are limited by differences in sampling schedules, assay platforms, drug structures, dosing regimens, study populations, and analytic definitions.

The mechanism underlying this IGF-1 increase remains incompletely defined. Reduced receptor-mediated ligand clearance after IGF-1R blockade and receptor internalization may decrease IGF-1 removal from the circulation (10). In addition, disruption of IGF-1R-mediated negative feedback within the GH–IGF-1 axis may promote compensatory GH release and hepatic IGF-1 production (14), a possibility supported by figitumumab data showing concurrent increases in GH and IGF-1 after IGF-1R blockade (12). However, because GH, IGF-binding proteins, drug concentrations, and receptor occupancy were not systematically assessed in our study, mechanisms interpretation remains inferential.

Our study further defines the timing and reversibility of IGF-1 elevation in routine TED treatment. Prior early-phase studies showed that serum IGF-1 could increase rapidly within 24–72 hours after IGF-1R inhibitor exposure (11, 12). In our cohort, the early increase was already pronounced before the second or third infusion-based assessment, and most patients approached their individual peak early during treatment. This timing is clinically plausible given the initial loading dose followed by higher maintenance doses and the prolonged half-life and sustained target engagement expected with teprotumumab treatment (10). After treatment cessation, IGF-1 declined gradually, with levels generally approaching baseline by 6–9 months or later. This recovery pattern appeared slower than would be expected from systemic antibody elimination alone, given the estimated teprotumumab half-life of approximately 20 days (10). One possible explanation is that IGF-1 normalization may also depend on waning residual receptor blockade and tissue-level target engagement, recovery of cell-surface IGF-1R signaling, and re-establishment of GH–IGF-1 negative feedback. Descriptively, patients receiving 8 infusions appeared to have more prolonged IGF-1 elevation than those receiving 4 infusions, but this observation should be considered exploratory because treatment duration was not randomized for this purpose and post-treatment sampling was incomplete.

Hyperglycemia is a recognized adverse effect of teprotumumab and other IGF-1R-directed therapies. Prior pooled trial and real-world data have shown that glycemic worsening occurs more frequently among patients with baseline prediabetes, diabetes, older age, or other features of metabolic vulnerability (15, 16, 20, 21). Mechanistically, this effect may reflect several overlapping pathways, including inhibition of IGF-1R-mediated insulin-sensitizing activity, interference with IGF-1R and insulin receptor signaling, and compensatory GH-related effects that may worsen insulin resistance (14, 21, 22). Consistent with these observations, HbA1c, GA, and FBG showed modest increases in our cohort, with greater glycemic deterioration among patients with baseline dysglycemia and other vulnerable clinical features. In addition, early changes were detectable in FBG and GA, highlighting the potential value of GA as a complementary marker because it reflects shorter-term glycemic variation than HbA1c and may capture early treatment-related changes more sensitively (23, 24).

A key finding of the present study is that circulating IGF-1 dynamics were not consistently identified as independent correlates of glycemic deterioration after accounting for baseline glycemic status and related clinical covariates. Beyond reflecting target engagement, measured serum IGF-1 did not appear to provide additional information on metabolic vulnerability, glycemic compensatory capacity, or individual susceptibility of the GH–IGF-1 axis. Although IGF-1 fold changes were lower in patients with worse baseline glycemic status, this difference may have been influenced by age, baseline metabolic status, or other clinical factors and did not translate into consistent independent associations with glycemic outcomes after adjustment. Therefore, circulating IGF-1 should not be used as a predictor or monitoring surrogate for hyperglycemia during teprotumumab N01 therapy.

Notably, an apparent delayed post-treatment IGF-1 peak or prolonged elevation was observed in patients with poorer baseline glycemic status, particularly those of diabetes group. However, this observation was based on limited post-treatment observations and a small subgroup sample. Whether this pattern reflects delayed pharmacodynamic recovery, persistent feedback adaptation, or slower metabolic compensation requires further study.

The main strength of this study is its longitudinal characterization of circulating IGF-1 during and after teprotumumab N01 therapy in a relatively large real-world TED cohort, a setting in which systematic clinical evidence remains limited. The use of infusion-based IGF-1 assessments, multiple glycemic markers, and additional analyses stratified by baseline glycemic status allowed a more clinically relevant evaluation of the relationship between IGF-1 dynamics and glycemic deterioration.

Several limitations should be acknowledged. First, the retrospective design limited control over sampling intervals and follow-up intensity, and post-treatment measurements were available only in a subset of patients, which may have introduced selection bias. Second, key mechanistic markers, including GH, IGF-binding proteins, insulin, C-peptide, and drug concentrations, were not systematically assessed, limiting the mechanistic interpretation. Third, although multivariable and subgroup analyses were performed, residual confounding by adiposity or other relevant metabolic factors cannot be excluded. Fourth, this was a single-center cohort including only Chinese patients, and the findings require validation in external and more diverse populations. Finally, whether IGF-1 dynamics are related to ophthalmic efficacy, durability of response, disease relapse, or other adverse events remains to be determined.

## Conclusions

In conclusion, teprotumumab N01 treatment was associated with a substantial, early, and broadly reversible increase in circulating IGF-1 in patients with moderate-to-severe TED, but IGF-1 dynamics did not serve as a reliable standalone indicator of glycemic deterioration during treatment.

## Data Availability

The study data could be available on reasonable request from the corresponding authors. Requests to access the datasets should be directed to XYZ, zhangxiuying717@163.com; LJ, jiln@bjmu.edu.cn.
