# Supplementary material for: Marked and reversible circulating insulin-like growth factor-1 elevation during teprotumumab N01 treatment for thyroid eye disease with limited correspondence to glycemic changes
Source: Front Endocrinol (Lausanne). 2026 Jul 15;17:1870933. doi: 10.3389/fendo.2026.1870933 (PMC13414759; doi:10.3389/fendo.2026.1870933)
Supplement: Supplementary file 1 [file DataSheet1.pdf]

# Supplemental Material

**Supplement to:** Wei Zhao, et al. Marked and reversible circulating insulin-like growth factor-1 elevation during teprotumumab N01 treatment for thyroid eye disease with limited correspondence to glycemic changes

**Table of Contents**


| Item | Content | | Page |
|---|---|---|---|
| 1 | Figure S1 | Serum IGF-1 dynamics stratified by sex | 2 |
| 2 | Figure S2 | Serum IGF-1 dynamics stratified by age group using a 45-year cutoff. | 3 |
| 3 | Figure S3 | Serum IGF-1 dynamics stratified by three age categories | 4 |
| 4 | Figure S4 | Longitudinal glycemic marker trajectories during and after treatment | 5 |
| 5 | Table S1 | Associations between IGF-1 dynamic metrics and continuous glycemic changes | 6–7 |
| 6 | Table S2 | Associations between IGF-1 dynamic metrics and glycemic events during treatment | 8–9 |
| 7 | Table S3 | Baseline HbA1c quartile-stratified characteristics among patients without diabetes history | 10 |
| 8 | Table S4 | Baseline HbA1c quartile-stratified associations between IGF-1 fold changes and peak glycemic changes among patients without diabetes history | 11 |

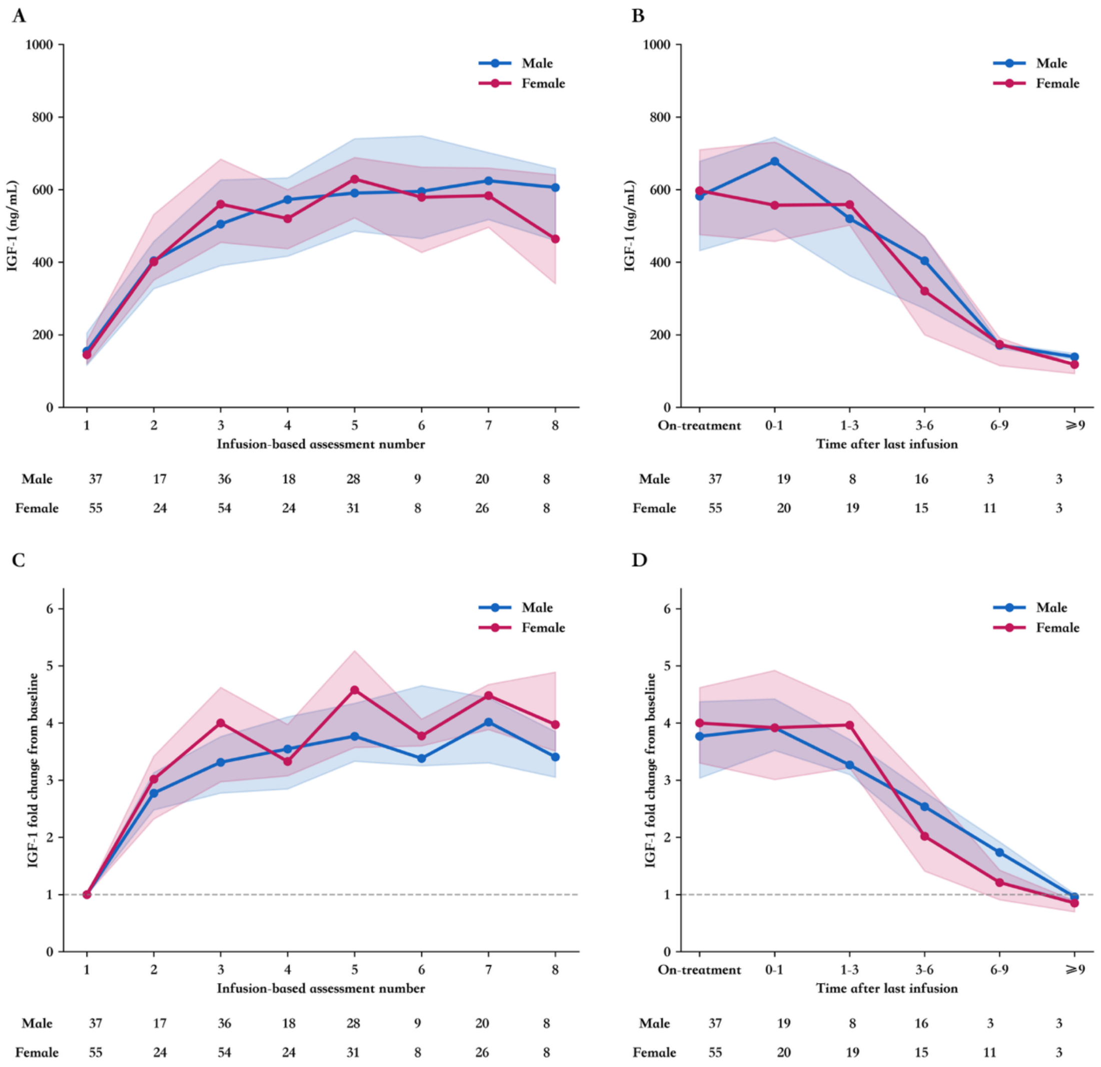


**Figure S1. Serum IGF-1 dynamics stratified by sex.** (A–B) Serum IGF-1 concentrations during treatment and post-treatment follow-up stratified by sex. (C–D) IGF-1 fold change from baseline stratified by sex. Lines and shaded areas indicate the median and interquartile range. The horizontal dashed line in panels C and D indicates a fold change of 1.0. The numbers below the x-axis indicate the number of patients with available data at each timepoint. IGF-1, insulin-like growth factor-1; IQR, interquartile range.

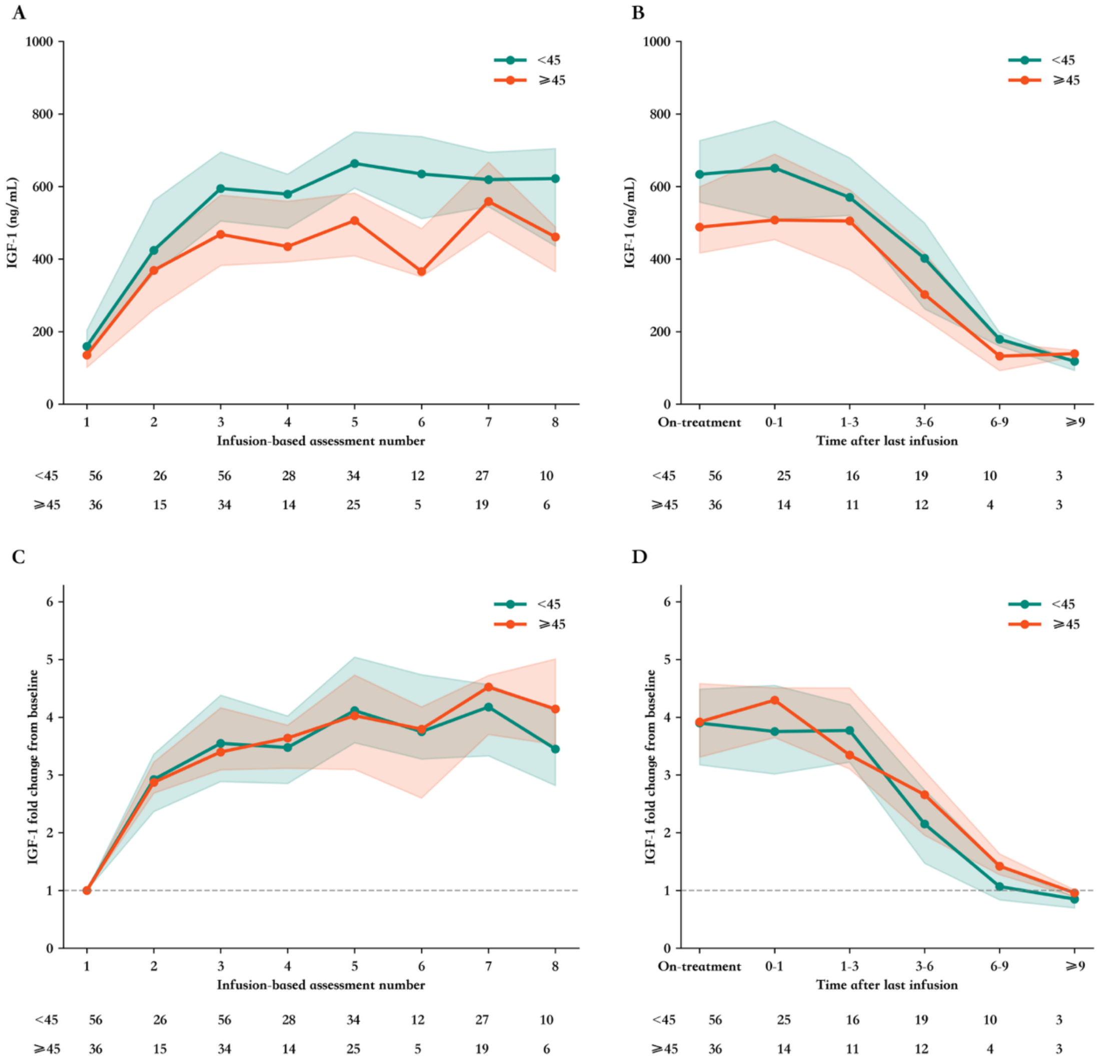


**Figure S2. Serum IGF-1 dynamics stratified by age group using a 45-year cutoff.** (A–B) Serum IGF-1 concentrations in patients aged (A) <45 years and (B) ≥45 years during treatment and post-treatment follow-up. (C–D) IGF-1 fold change from baseline in patients aged (C) <45 years and (D) ≥45 years. Lines and shaded areas indicate the median and interquartile range. The horizontal dashed line in panels C and D indicates a fold change of 1.0. The numbers below the x-axis indicate the number of available data at each timepoint. IGF-1, insulin-like growth factor-1; IQR, interquartile range.

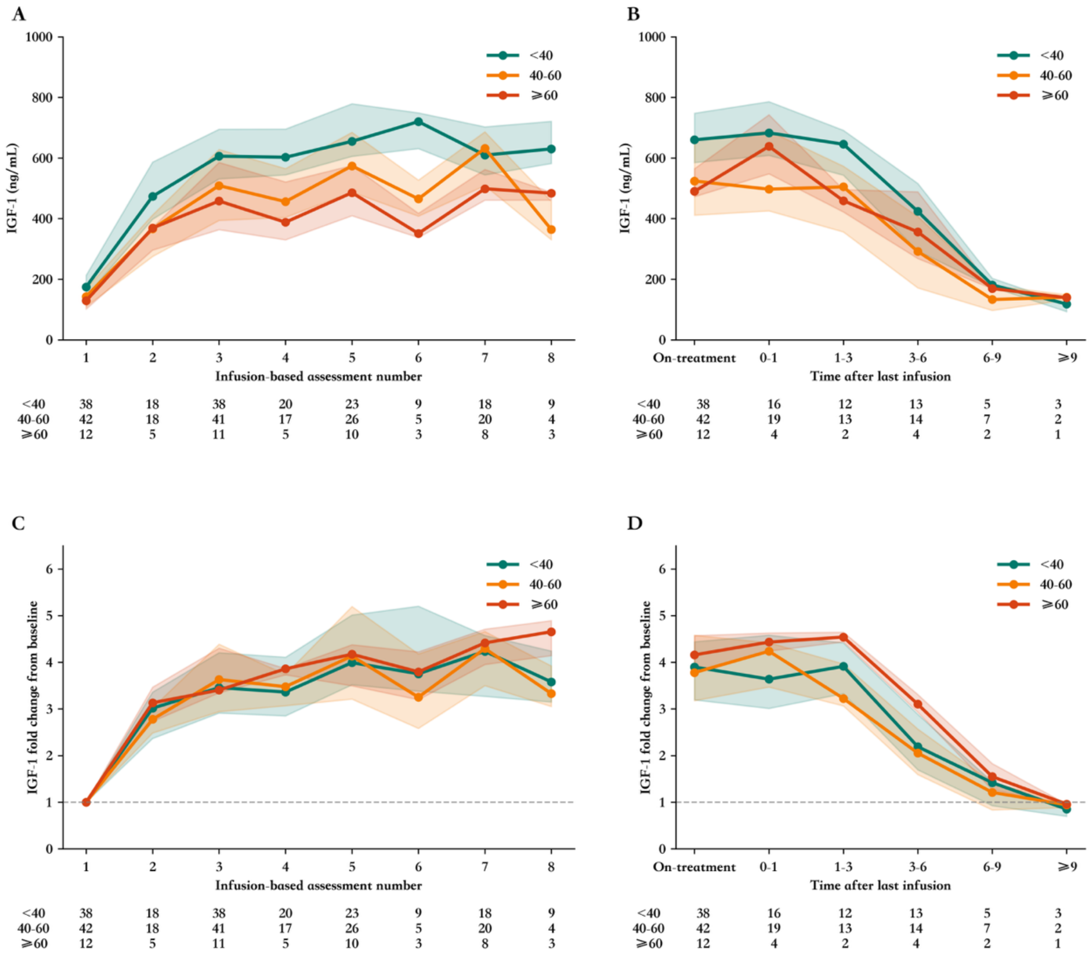


**Figure S3. Serum IGF-1 dynamics stratified by three age categories.** (A–B) Serum IGF-1 concentrations during treatment and post-treatment follow-up stratified by age group (<40, 40–60, and ≥60 years). (C–D) IGF-1 fold change from baseline stratified by the same age groups. Lines and shaded areas indicate the median and interquartile range. The horizontal dashed line in panels C and D indicates a fold change of 1.0. The numbers below the x-axis indicate the number of available data at each timepoint. IGF-1, insulin-like growth factor-1; IQR, interquartile range.

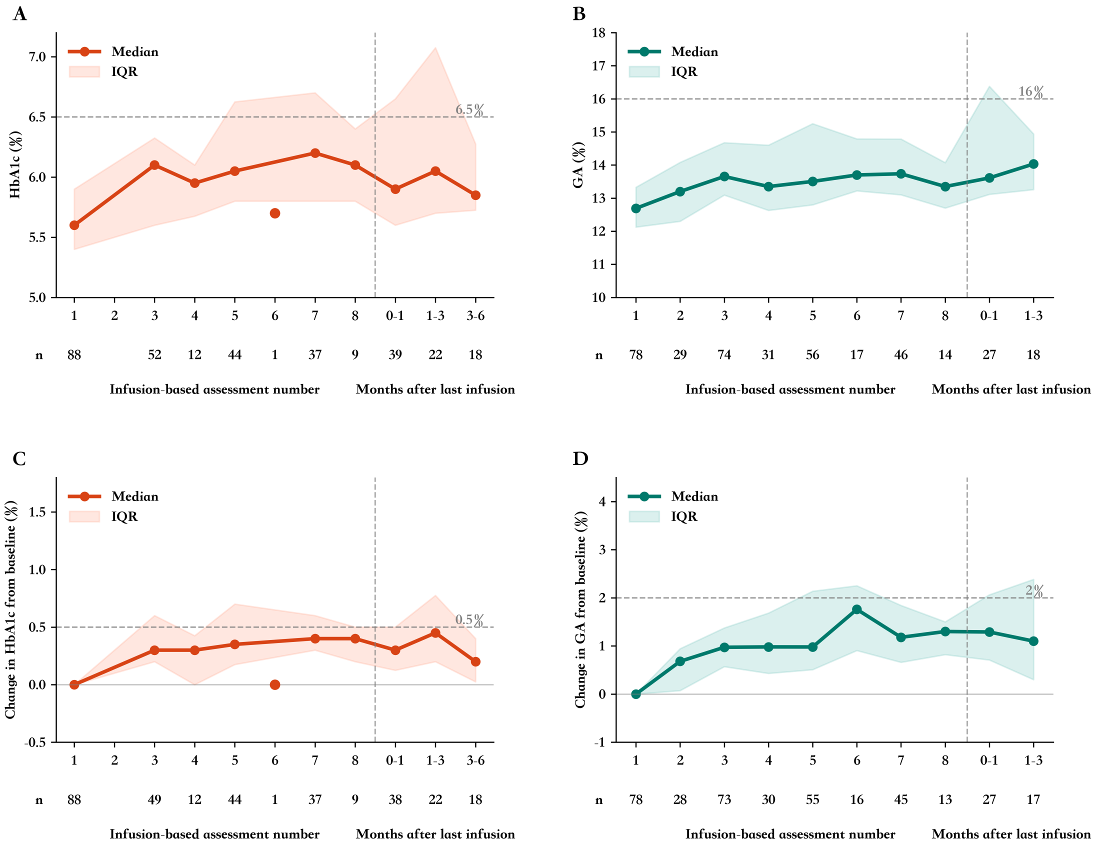


**Figure S4. Longitudinal glycemic marker trajectories during and after treatment.** (A) HbA1c and (B) GA values during treatment and post-treatment follow-up. Change in (C) HbA1c and (D) GA from baseline during treatment and post-treatment follow-up. Points with only one available observation were displayed but excluded from line and IQR interpolation. The numbers below the x-axis indicate the number of patients with available data at each timepoint. HbA1c, hemoglobin A1c; IQR, interquartile range.

**Table S1. Associations between IGF-1 dynamic metrics and continuous glycemic changes**

| Glycemic outcome | IGF-1 variable | N | Model 1 | | Model 2 | | Model 3 | |
|---|---|---|---|---|---|---|---|---|
| | | | β (95% CI) | P | β (95% CI) | P | β (95% CI) | P |
| Early ΔHbA1c | Baseline IGF-1 | 49 | 0.09 (-0.22, 0.40) | 0.553 | 0.09 (-0.19, 0.36) | 0.544 | 0.09 (-0.19, 0.36) | 0.544 |
| Early ΔHbA1c | Early IGF-1 fold | 47 | -0.16 (-0.47, 0.15) | 0.299 | 0.01 (-0.27, 0.29) | 0.964 | 0.05 (-0.19, 0.29) | 0.673 |
| Early ΔGA | Baseline IGF-1 | 76 | -0.05 (-0.30, 0.19) | 0.671 | -0.05 (-0.29, 0.18) | 0.666 | -0.05 (-0.29, 0.18) | 0.666 |
| Early ΔGA | Early IGF-1 fold | 76 | -0.10 (-0.33, 0.12) | 0.370 | -0.13 (-0.37, 0.12) | 0.302 | -0.18 (-0.45, 0.09) | 0.186 |
| Early ΔFBG | Baseline IGF-1 | 90 | -0.02 (-0.20, 0.16) | 0.819 | -0.04 (-0.21, 0.13) | 0.662 | -0.04 (-0.21, 0.13) | 0.662 |
| Early ΔFBG | Early IGF-1 fold | 88 | -0.12 (-0.35, 0.11) | 0.310 | -0.07 (-0.27, 0.12) | 0.455 | -0.10 (-0.33, 0.13) | 0.394 |
| Peak ΔHbA1c | Baseline IGF-1 | 74 | 0.11 (-0.14, 0.36) | 0.405 | 0.08 (-0.14, 0.29) | 0.490 | 0.08 (-0.14, 0.29) | 0.490 |
| Peak ΔHbA1c | Early IGF-1 fold | 72 | -0.12 (-0.39, 0.16) | 0.414 | 0.09 (-0.13, 0.32) | 0.402 | 0.15 (-0.05, 0.36) | 0.138 |
| Peak ΔHbA1c | Peak IGF-1 fold | 74 | -0.11 (-0.35, 0.13) | 0.368 | 0.05 (-0.15, 0.24) | 0.639 | 0.12 (-0.08, 0.33) | 0.237 |
| Peak ΔGA | Baseline IGF-1 | 78 | -0.06 (-0.27, 0.15) | 0.572 | -0.06 (-0.26, 0.14) | 0.530 | -0.06 (-0.26, 0.14) | 0.530 |
| Peak ΔGA | Early IGF-1 fold | 77 | -0.17 (-0.43, 0.09) | 0.198 | -0.19 (-0.46, 0.08) | 0.171 | -0.26 (-0.58, 0.05) | 0.103 |
| Peak ΔGA | Peak IGF-1 fold | 78 | -0.11 (-0.36, 0.14) | 0.386 | -0.12 (-0.37, 0.13) | 0.362 | -0.22 (-0.58, 0.13) | 0.215 |
| Peak ΔFBG | Baseline IGF-1 | 90 | 0.02 (-0.18, 0.21) | 0.875 | 0.00 (-0.19, 0.19) | 0.994 | 0.00 (-0.19, 0.19) | 0.994 |
| Peak ΔFBG | Early IGF-1 fold | 88 | -0.12 (-0.36, 0.12) | 0.335 | -0.08 (-0.28, 0.12) | 0.446 | -0.08 (-0.32, 0.15) | 0.485 |

| | | | | | | | | |
|---|---|---|---|---|---|---|---|---|
| Peak ΔFBG | Peak IGF-1 fold | 90 | -0.07 (-0.32, 0.18) | 0.591 | -0.03 (-0.24, 0.19) | 0.822 | -0.04 (-0.34, 0.27) | 0.821 |

Values are standardized β coefficients with 95% confidence intervals and nominal P values from linear regression models. Model 1 was adjusted for age and sex. Model 2 was adjusted for age, sex, and the corresponding baseline glycemic marker. Model 3 was adjusted for age, sex, the corresponding baseline glycemic marker, and baseline IGF-1 when applicable. For models in which baseline IGF-1 was the exposure, baseline IGF-1 was not additionally included as a covariate. IGF-1 fold change was calculated relative to baseline IGF-1. FBG, fasting blood glucose; GA, glycated albumin; HbA1c, hemoglobin A1c; IGF-1, insulin-like growth factor-1; CI, confidence interval.

**Table S2. Associations between IGF-1 dynamic metrics and glycemic events during treatment**

| Glycemic event | IGF-1 variable | Events / N | Model 1 | | Model 2 | | Model 3 | |
|---|---|---|---|---|---|---|---|---|
| | | | OR (95% CI) | P | OR (95% CI) | P | OR (95% CI) | P |
| Peak HbA1c ≥6.5% | Baseline IGF-1 | 20/72 | 1.26 (0.71–2.23) | 0.424 | 1.08 (0.46–2.52) | 0.864 | 1.08 (0.46–2.52) | 0.864 |
| Peak HbA1c ≥6.5% | Early IGF-1 fold | 20/72 | 0.47 (0.23–0.96) | 0.037 | 1.02 (0.41–2.52) | 0.970 | 1.06 (0.39–2.87) | 0.904 |
| Peak HbA1c ≥6.5% | Peak IGF-1 fold | 20/72 | 0.40 (0.19–0.84) | 0.015 | 0.71 (0.30–1.70) | 0.449 | 0.64 (0.22–1.86) | 0.413 |
| Δpeak HbA1c ≥0.5% | Baseline IGF-1 | 30/72 | 1.22 (0.72–2.07) | 0.450 | 1.20 (0.67–2.14) | 0.536 | 1.20 (0.67–2.14) | 0.536 |
| Δpeak HbA1c ≥0.5% | Early IGF-1 fold | 30/72 | 0.75 (0.44–1.29) | 0.303 | 1.05 (0.57–1.93) | 0.880 | 1.14 (0.60–2.18) | 0.691 |
| Δpeak HbA1c ≥0.5% | Peak IGF-1 fold | 30/72 | 0.70 (0.41–1.20) | 0.195 | 0.91 (0.52–1.61) | 0.754 | 1.01 (0.51–1.98) | 0.979 |
| Peak GA ≥16% | Baseline IGF-1 | 16/77 | 1.00 (0.53–1.89) | 0.992 | 1.00 (0.48–2.07) | 0.993 | 1.00 (0.48–2.07) | 0.993 |
| Peak GA ≥16% | Early IGF-1 fold | 16/77 | 1.04 (0.54–1.97) | 0.914 | 0.94 (0.46–1.93) | 0.868 | 0.93 (0.43–2.00) | 0.857 |
| Peak GA ≥16% | Peak IGF-1 fold | 16/77 | 1.03 (0.57–1.87) | 0.917 | 1.06 (0.54–2.07) | 0.872 | 1.08 (0.49–2.37) | 0.855 |
| Δpeak GA ≥2% | Baseline IGF-1 | 28/77 | 0.85 (0.49–1.46) | 0.547 | 0.84 (0.49–1.46) | 0.545 | 0.84 (0.49–1.46) | 0.545 |
| Δpeak GA ≥2% | Early IGF-1 fold | 28/77 | 0.73 (0.43–1.26) | 0.262 | 0.72 (0.41–1.25) | 0.240 | 0.58 (0.30–1.13) | 0.108 |
| Δpeak GA ≥2% | Peak IGF-1 fold | 28/77 | 0.85 (0.52–1.41) | 0.539 | 0.85 (0.51–1.41) | 0.531 | 0.67 (0.35–1.28) | 0.225 |

Values are odds ratios with 95% confidence intervals and nominal P values from logistic regression models. Model 1 was adjusted for age and sex. Model 2 was adjusted for age, sex, and the corresponding baseline glycemic marker. Model 3 was adjusted for age, sex, the corresponding baseline glycemic marker, and baseline IGF-1 when applicable. For models in which baseline IGF-1 was the exposure, baseline IGF-1 was not additionally

included as a covariate. IGF-1 fold change was calculated relative to baseline IGF-1. GA, glycated albumin; HbA1c, hemoglobin A1c; IGF-1, insulin-like growth factor-1; OR, odds ratio; CI, confidence interval.

**Table S3. Baseline HbA1c quartile-stratified characteristics among patients without diabetes history**

| Characteristic | HbA1c Q1 (N=23) | HbA1c Q2 (N=22) | HbA1c Q3 (N=22) | HbA1c Q4 (N=16) |
|---|---|---|---|---|
| Baseline HbA1c range (%) | 5.10–5.40 | 5.50–5.60 | 5.70–5.90 | 6.00–6.90 |
| Baseline HbA1c (%) | 5.30 (5.20, 5.40) | 5.60 (5.53, 5.60) | 5.80 (5.70, 5.90) | 6.20 (6.00, 6.33) |
| Baseline FBG (mmol/L) | 4.69 (4.56, 5.01) | 4.75 (4.63, 5.11) | 4.92 (4.70, 5.10) | 4.93 (4.58, 5.56) |
| Baseline GA (%) | 12.52 (12.11, 13.24) | 12.81 (12.11, 13.31) | 12.38 (12.11, 12.70) | 12.91 (12.80, 14.08 |
| Baseline IGF-1 (ng/mL) | 157.00 (114.50, 206.50) | 141.00 (114.00, 158.00) | 157.50 (105.25, 192.25) | 148.00 (133.00, 191.50) |
| Early IGF-1 fold change | 3.91 (3.29, 5.09) | 4.38 (3.76, 4.54) | 3.20 (2.80, 3.40) | 3.26 (2.86, 3.80) |
| Peak IGF-1 fold change | 4.55 (3.69, 5.63) | 4.77 (4.53, 5.19) | 3.80 (3.46, 4.47) | 3.74 (3.06, 4.57) |
| Early FBG change (mmol/L) | 0.07 (-0.37, 0.37) | 0.33 (0.01, 0.74) | 0.33 (-0.13, 0.72) | 0.65 (0.32, 1.21) |
| Early HbA1c change (%) | 0.20 (0.15, 0.40) | 0.30 (0.20, 0.60) | 0.35 (0.15, 0.40) | 0.45 (0.08, 0.92) |
| Early GA change (%) | 0.70 (0.33, 0.92) | 1.13 (0.64, 1.44) | 1.16 (0.90, 1.34) | 1.30 (0.74, 1.74) |
| Peak FBG change (mmol/L) | 0.35 (-0.02, 0.58) | 0.63 (0.43, 1.04) | 0.68 (0.32, 1.21) | 1.00 (0.76, 2.10) |
| Peak HbA1c change (%) | 0.35 (0.05, 0.40) | 0.40 (0.30, 0.70) | 0.30 (0.10, 0.48) | 0.90 (0.55, 1.25) |
| Peak change≥0.5% [n/N (%)] | 3/18 (16.7) | 11/22 (50.0) | 5/18 (27.8) | 8/11 (72.7) |
| Peak GA change (%) | 0.83 (0.53, 1.22) | 1.28 (1.17, 1.96) | 1.74 (1.21, 2.01) | 2.79 (2.24, 3.52) |
| Peak change≥2% [n/N (%)] | 1/19 (5.3) | 5/20 (25.0) | 6/18 (33.3) | 12/14 (85.7) |

Data are shown among patients without documented diabetes history and with available baseline HbA1c values. HbA1c quartiles were generated within this subgroup. Continuous variables are presented as median (interquartile range), and categorical outcomes are presented as n/N (%) among patients with available data. FBG, fasting blood glucose; GA, glycated albumin; HbA1c, hemoglobin A1c; IGF-1, insulin-like growth factor-1.

**Table S4. Baseline HbA1c quartile-stratified associations between IGF-1 fold changes and peak glycemic changes among patients without diabetes history**

| Outcome | IGF-1 variable | HbA1c Q1 | | HbA1c Q2 | | HbA1c Q3 | | HbA1c Q4 | |
|---|---|---|---|---|---|---|---|---|---|
| | | β (95% CI) | P | β (95% CI) | P | β (95% CI) | P | β (95% CI) | P |
| Peak ΔHbA1c | Early IGF-1 fold | -0.09 (-0.58, 0.40) | 0.712 | 0.10 (-0.79, 0.98) | 0.832 | 0.22 (-0.22, 0.66) | 0.333 | -0.83 (-2.17, 0.51) | 0.224 |
| Peak ΔHbA1c | Peak IGF-1 fold | -0.25 (-0.91, 0.40) | 0.451 | 0.33 (-1.19, 1.85) | 0.669 | 0.07 (-0.66, 0.81) | 0.841 | -0.26 (-1.82, 1.29) | 0.741 |
| Peak ΔGA | Early IGF-1 fold | -0.08 (-0.69, 0.53) | 0.800 | 0.08 (-1.06, 1.21) | 0.893 | 0.34 (-0.37, 1.05) | 0.344 | -1.07 (-2.02, -0.12) | 0.028 |
| Peak ΔGA | Peak IGF-1 fold | 0.09 (-0.52, 0.70) | 0.769 | 0.13 (-1.62, 1.87) | 0.884 | 0.18 (-0.54, 0.90) | 0.626 | -0.47 (-1.76, 0.83) | 0.478 |
| Peak ΔFBG | Early IGF-1 fold | 0.10 (-0.21, 0.40) | 0.532 | 0.07 (-0.82, 0.96) | 0.876 | 0.09 (-0.36, 0.54) | 0.701 | -0.66 (-2.64, 1.32) | 0.511 |
| Peak ΔFBG | Peak IGF-1 fold | 0.19 (-0.08, 0.45) | 0.161 | 0.24 (-1.07, 1.55) | 0.723 | 0.15 (-0.24, 0.55) | 0.448 | -0.25 (-2.11, 1.60) | 0.789 |

Values are standardized β coefficients with 95% confidence intervals and nominal P values from Model 3 linear regression analyses. Model 3 was adjusted for age, sex, the corresponding baseline glycemic marker, and baseline IGF-1. HbA1c quartiles were generated among patients without documented diabetes history and with available baseline HbA1c values. IGF-1 fold change was calculated relative to baseline IGF-1. FBG, fasting blood glucose; GA, glycated albumin; HbA1c, hemoglobin A1c; IGF-1, insulin-like growth factor-1; CI, confidence interval.
